# The Impact of Government Official Assessment on Ecological Poverty Alleviation: Evidence from Chinese Listed Companies

**DOI:** 10.3390/ijerph19063470

**Published:** 2022-03-15

**Authors:** Yujing Huang

**Affiliations:** Economics and Management School, Wuhan University, Wuhan 430072, China; joycehuang@whu.edu.cn

**Keywords:** ecological poverty alleviation, environmental governance, corporate social responsibility, official assessment

## Abstract

Ecological poverty alleviation launched by the Chinese government is an innovative green development measure that combines targeted poverty alleviation with ecological protection to realize the ecological environmental protection and income growth of the impoverished population. Based on the Chinese government’s policy of poverty alleviation assessment for provincial government officials in 2016, this paper studies whether the assessment of government officials promote enterprises’ participation in ecological poverty alleviation. Using the sample of Chinese A-share listed companies from 2016 to 2020, the empirical test shows that the more important the assessment of poverty alleviation by officials, the more likely local enterprises are to participate in targeted poverty alleviation and the higher the investment level is likely to be. The results pass a series of robustness tests. In addition, this paper further finds that enterprise participation in ecological poverty alleviation can effectively reduce local water pollution, air pollution and solid pollution, thus improving the ecological environment. It suggests that the assessment mechanism of Chinese government officials can effectively promote multi-dimensional ecological poverty alleviation. The contributions of this paper are as follows. Firstly, it is helpful to expand the relevant literature on enterprise environmental protection from the perspective of ecological poverty alleviation. Secondly, it is helpful to expand the literature related to government–enterprise interaction from the perspective of the assessment of government officials. Finally, it is helpful to enrich and expand the relevant literatures on promotion incentives of government officials from the perspective of ecological poverty alleviation.

## 1. Introduction

The construction of ecological civilization has become an inevitable requirement for the country to accelerate the transformation of economic development and achieve green development [1,2]. In recent years, the Chinese government has attached great importance to energy conservation, emission reduction and ecological environment improvement, making a major contribution to global ecological governance. In 2019, the United Nations Environment Program evaluated *A Review of 20 Years’ Air Pollution Control in Beijing*, emphasizing that Beijing has achieved remarkable results in the improvement of air environment quality and that China’s experience is worth learning from other countries [3]. It is closely related to the official governance mode under the political centralization in China [4,5].

In China, as a dual battleground for poverty alleviation and ecological protection, poor areas in the central and western regions should prioritize restoring the ecological environment [6,7] and explore effective ways of implementing ecological poverty alleviation. The State Council’s *The Thirteenth Five-Year Plan for Poverty Alleviation*, released in November 2016, emphasizes that ecological poverty alleviation nicely coordinates ecological protection with poverty alleviation. Specifically, ecological poverty alleviation breaks the vicious circle of ecological destruction and poverty by strengthening resource protection, ecological management and restoration in poor areas, and enhancing the sustainable development capacity of poor areas. It achieves a win–win situation for both the ecological environment and poverty alleviation and development [8].

Ecological poverty alleviation has been carried out nationwide in China. Governments have mobilized social forces to participate in it, with enterprises being an important force [9]. In contrast, enterprises only participate in public governance through donation [10,11], enterprises can improve the efficiency of ecological poverty alleviation by directly participating in various forms, such as manpower, materials and funds. As a special form of corporate social responsibility, the motivation of firms to participate in ecological poverty alleviation needs to be discussed in depth.

Ecological poverty alleviation, as a special form of corporate social responsibility, has different motivations for its actions. The General Office of the CPC Central Committee and The General Office of the State Council jointly issued *The Measures for Assessing the Effectiveness of Poverty Alleviation and Development Work of Provincial Party Committees and Governments* in February 2016. Provincial government officials are regularly evaluated based on ecological poverty alleviation. The assessment result is an important basis for the comprehensive assessment and evaluation of provincial government officials. Local government officials have a strong need for promotion and will do their utmost to meet promotion assessment targets [12,13,14]. Therefore, local government officials, while playing a leading role in investing resources in ecological poverty alleviation, have an incentive to fully guide firms to participate in it through their strong social mobilization capacity, forming a joint effort between government and firms. In addition, firms are interested in implementing ecological poverty alleviation together with local governments, which helps to form good interaction with local governments [15,16]. Therefore, this paper tests the motivation of firms to participate in ecological poverty alleviation from the perspective of the importance of ecological poverty alleviation assessment to the government official. This paper uses hand-collected data on the ecological poverty alleviation of A-share listed companies from 2016 to 2020 to test the above hypotheses and finds that firms are more likely and have a higher level of commitment to ecological poverty alleviation in central and western regions, compared to eastern regions. Firms in regions with greater poverty are more likely to participate in ecological poverty alleviation and have higher levels of investment than firms in regions with less poverty. This paper further examines the economic consequences of poverty alleviation and finds that the participation of enterprises in poverty alleviation reduces the level of local pollution.

The academic contributions of this paper are as follows.

Firstly, this paper helps to expand the research on corporate environmental protection from the perspective of ecological poverty alleviation. Existing works in the literature mainly study the influencing factors of corporate environmental protection from the aspects of corporate reputation [17], and public opinion pressure [18,19]. Additionally, existing works in the literature study the benefits of environmental protection to enterprises from the perspective of improving market performance [20,21], reducing enterprise risk [22] and non-linear impact on financial performance [23]. However, there are few studies on ecological poverty alleviation as a special way of environmental protection. Ecological poverty alleviation refers to a green poverty alleviation concept and method that combines targeted poverty alleviation with ecological protection. This paper directly studies a new and special environmental protection behavior, which can enrich and expand the relevant research on green investment from the micro level and per se contributes to our general understanding of the role of corporate social responsibility [24,25,26,27].

Secondly, this paper is helpful to expand the literature related to the government–enterprise interaction from the perspective of the assessment of government officials. As for the relevant literature on the government–enterprise interaction, the existing works mainly identify the policy burden of state-owned enterprises through government intervention [28,29] and show that while firms help politicians achieve their personal political goals, politicians also give benefits back [30,31,32]. For example, Lin et al. [10] found that firms use CSR to build political networks and are rewarded with more government subsidies. Bertrand et al. [33] found that corporate philanthropy acts as tax-exempt lobbying for firms to exert political influence. The process of government–enterprise interaction studied in this paper is based on the assessment of local government officials as a logical starting point. Because local politicians are rigorously assessed for their effectiveness in poverty alleviation, the results are linked to their promotions. Local politicians have an incentive to force enterprises to participate in ecological poverty alleviation to achieve their political goals. This is clearly about the role of the assessment mechanism of local government officials, compared to previous works in the literature. Therefore, this paper provides new evidence for relevant literature on the government–enterprise interaction from the perspective of government officials’ assessment.

Finally, this paper helps to enrich the relevant literature on promotion incentives for government officials from the perspective of ecological poverty alleviation. Existing studies on promotion incentives for government officials mostly focus on the impact of promotion incentives on regional economic growth [34,35], bank bankruptcy [36,37], investment scale [38,39] and firm performance [40,41,42,43]. However, few works study the influence of promotion incentive mechanism on corporate social activities. This paper finds that the official assessment mechanism can promote enterprises to participate in ecological poverty alleviation, which indicates that the official assessment can stimulate not only the economic decisions, but also non-economic decisions. Therefore, this paper enriches the relevant literature on the promotion incentives of government officials from the perspective of ecological poverty alleviation.

In addition, the findings of this paper illustrate that the officials’ poverty alleviation supervision and assessment system has effectively promoted ecological poverty alleviation. Therefore, the research in this paper has reference value and implications for improving the assessment and evaluation work in government governance and provides a theoretical basis and policy reference for comprehensively promoting rural revitalization and green development.

The rest of the paper is organized as follows: Section 2 contains the institutional background and research hypotheses. Section 3 shows the research methodology. Section 4 presents the empirical results. Section 5 gives the conclusion, limitations, and further discussion.

## 2. Institutional Background and Research Hypotheses

### 2.1. Institutional Background

#### 2.1.1. Ecological Poverty Alleviation Policy System

Previous works in the literature suggest that environmental governance and poverty reduction interact. Brockington and Schmidt-Soltau [44] found that environmental conservation measures result in greater local impoverishment by limiting people’s access to natural resources. Alix-garcia et al. [45] found that the increase in the income of the poor might aggravate deforestation. Alix-garcia et al. [46] found that cash transfer plan implemented in Mexico can reduce land cover loss and produce small-scale but positive poverty alleviation effect. China has tried to combine increasing the income of the impoverished population with improving the ecological environment, introducing ecological poverty alleviation programs [8]. On 23 November 2016, The State Council issued *The Thirteenth Five-Year Plan for Poverty Alleviation*, which focuses on handling the relationship between ecological protection and poverty alleviation and development, strengthening ecological environmental protection, governance and restoration in poverty-stricken areas, and enhancing the sustainable development capacity of poverty-stricken areas. In 2018, the Ministry of Ecology and Environment of China issued *The Guiding Opinions on Ecological and Environmental Protection to Help Win the Tough Battle against Targeted Poverty alleviation*, which put forward specific suggestions on strengthening ecological and environmental protection for poverty alleviation. Impoverished areas should take advantage of local environment conditions, regulate the planting and breeding industry and rural tourism industry, increase ecological protection and restoration in poor areas, and create jobs related to ecological protection for impoverished people.

#### 2.1.2. Appraisal and Evaluation System for Government Officials

China’s political centralization plays an important role in economic and social development [4]. The central government manages local government officials through performance-related assessments to reduce agency costs [34]. Previous works in the literature found that official promotion incentive can promote local investment [47], increase credit expansion [48] and improve water pollution [49]. As poverty alleviation strategies are becoming more important, the Chinese government have begun to evaluate the effectiveness of local government officials in poverty alleviation. Specifically, the General Office of the CPC Central Committee and The General Office of the State Council jointly issued *The Measures for Assessing the Effectiveness of Poverty Alleviation and Development Work of Provincial Party Committees and Governments* in February 2016. The assessment method applies to government officials in 22 provinces in Central and Western China. The State Council Leading Group for Poverty Alleviation and Development organizes assessment annually. The work designs assessment indicators for targeted poverty alleviation tasks and summarizes the results into a comprehensive evaluation. The assessment includes the poverty reduction effect of various poverty alleviation projects, people’s satisfaction with the work, and the management of poverty alleviation funds. Among them, indicators related to ecological poverty alleviation, such as resource consumption, environmental damage, and increase and decrease in ecological benefits, are included in the evaluation system, and the assessment weight is increased. Therefore, the ecological poverty alleviation results are very important for the promotion of provincial government officials [50].

#### 2.1.3. Corporate Approach to Ecological Poverty Alleviation

Previous works in the literature showed that enterprises in environmentally sensitive industries will fulfill environment-related corporate social responsibility and increase green investment due to pressure from stakeholders [51,52,53]. *The Thirteenth Five-Year Plan for Poverty Alleviation* emphasizes that local governments should actively mobilize enterprises to play a complementary role in ecological poverty alleviation. In ecological poverty alleviation, enterprises can give full play to their advantages in capital, management and human resources [8] through mature environmental management technology and improving the efficiency of government environmental governance [54,55]. Enterprises can develop market-oriented ecological industries, such as eco-agriculture and its processing industry, eco-tourism and photovoltaic industry, to lift the poor out of poverty.

### 2.2. Research Hypothesis

Under the Chinese style of decentralization, Chinese government officials have been in a closed bureaucracy for a long time, and once they enter the bureaucracy, they must strive to retain their positions and seek all possible opportunities for promotion [56]. As a result, local government officials compete horizontally according to the performance appraisal indicators set by the central government [57,58]. The features amount to the regionally decentralized authoritarian system as termed in Xu [59]. The performance appraisal system for local government officials has changed from the initial purely political indicators to economic performance indicators with GDP growth as the core, and then gradually downplayed GDP indicators and emphasized the concept of green GDP. Additionally, along with the increasing prominence of precise poverty alleviation in the country’s governance, the effectiveness of poverty alleviation has also become an important indicator in the comprehensive evaluation of officials. As a result, the assessment of ecological poverty alleviation has an increasingly significant impact on the promotion of officials.

As stated in the theoretical model in Shleifer and Vishny [28], local government officials will do their utmost to mobilize human, material, and financial resources to fulfill the assessment targets when the effectiveness of ecological poverty alleviation is embedded into their political loyalty and career advancement goals. Therefore, while local government officials play a leading role in investing resources in poverty alleviation, they also have an incentive to mobilize enterprises to participate in poverty alleviation through their strong social mobilization power. It has been documented that it is common for enterprises to help local government officials achieve their political goals. In France, corporate CEOs with political backgrounds help re-elect politicians in their districts by having higher job and plant creation rates [60]. In transition and developing economies, governments intervene in the investment allocation tendencies of firms, thereby achieving government objectives [61] or stimulating economic growth by intervening in bank loans to finance projects [48]. While these papers focus on political goals centered on GDP, this paper attempts to examine the pro-poor political goals of local government officials.

It is hard for firms to remain indifferent to the programs that governments value. On the one hand, compared with private enterprises, state-owned enterprises are like a quasi-administrative organization that undertakes a large number of administrative and social responsibilities contracted by the government, leading to a greater tendency of “political catering” in state-owned enterprises [62]. *The Thirteenth Five-Year Plan for Poverty Alleviation* emphasizes that central enterprises should play a “leading and exemplary role”, “strengthen” the responsibility of state-owned enterprises to help, and “encourage” private enterprises to participate. It can be seen that the government has high hopes for the supporting role of state-owned enterprises. On the other hand, private enterprises will take ecological poverty alleviation as the preferred way to construct and maintain their political connections. To return the favor, politicians provide resources to firms to ‘reward’ the support from businessmen [63,64,65]. For example, firms that construct political connections may obtain more favorable contract amounts [16], incentives in the renegotiation of government contracts [66], obtain a cheaper price in land deals [15] and receive the benefit of government contracts [67,68,69]. Based on the above theoretical analysis, this paper argues that local government officials are likely to use their political relationships and strong social mobilization power to drive companies to participate in poverty alleviation. Firms are also likely to respond to national strategic calls and maintain good relations with the government by participating in poverty alleviation. However, even in the face of the same rules of assessment, the importance of poverty alleviation assessment faced by officials in different provinces is not the same in the comprehensive assessment, thus driving different incentives for firms to share the burden.

First, the Measures for Assessing the Effectiveness of Poverty Alleviation and Development Work of Provincial Party Committees and Governments only applies to government officials in 22 provinces in Central and Western China. Although the governments of eastern provinces also carry out poverty alleviation cooperation between the east and the west, the assessment results of poverty alleviation cooperation are not the basis for the comprehensive assessment of government officials in eastern provinces. Therefore, this paper believes that the assessment of poverty alleviation of government officials in 22 central and western provinces is more important than that of government officials in eastern provinces. Secondly, the greater the demand for poverty alleviation in a region, the greater the importance of poverty alleviation assessment faced by local government officials. In areas where poverty alleviation assessment is more important, local government officials are more likely to require enterprises to participate in targeted poverty alleviation to achieve assessment targets and improve the probability of promotion.

Therefore, based on the above analysis, this paper proposes the research hypothesis:

**Hypothesis** **1** **(H1).** *The more important the assessment of poverty alleviation by officials, the more likely local enterprises are to participate in targeted poverty alleviation, and the greater the input level is.*

## 3. Research Methodology

### 3.1. Sample and Data

In December 2016, the Shanghai Stock Exchange and Shenzhen Stock Exchange successively issued *The Notice on Further Improving Information Disclosure on Poverty Alleviation Work of Listed Companies*, which set out requirements for further improving information disclosure on poverty alleviation work. The listed companies separately list the annual ecological poverty alleviation work in the important matters section of their annual reports, which contains information on the targets, content, and amount of ecological poverty alleviation. Therefore, we can obtain quantitative information on the participation of enterprises in ecological poverty alleviation from the annual reports of listed companies to measure the willingness of firms to participate in ecological poverty alleviation.

This paper selects A-share listed companies in Shanghai and Shenzhen in China from 2016 to 2020 as the research sample, and after proposing the financial sector, ST companies, and the missing sample of variables. This paper finally obtains 11,527 valid samples. The ecological poverty alleviation data are manually collected from annual reports, while the rest of the financial data are obtained from the China Stock Market and Accounting Research Database (CSMAR) database, macroeconomic data from the National Bureau of Statistics, and national poverty counties data from the China Poverty Alleviation Database. To eliminate the influence of extreme samples on the results, the continuous variables related to firm characteristics are subject to bilateral winsorizing at 1% and 99% levels, annually.

### 3.2. Empirical Model

To test Hypothesis 1, the regression equation to be tested in this paper is set as follows:

(1) $$Povref_{i,t}=\alpha +\beta_{1}Midwest_{i,t}\left(Poor_{i,t}\right)+\beta_{2}Control_{i,t}+\varphi_{t}+\mu_{j}+\varepsilon$$

In Equation (1), *Povref* is a proxy variable for firm ecological poverty alleviation behavior, which is measured by using whether the firm participates in ecological poverty alleviation (*Povref_eco*) and the amount of ecological poverty alleviation inputs disclosed in the annual report plus one, and then taking the natural log (*Input_eco*), respectively. Operating income and total assets are also standardized for poverty alleviation inputs to measure the ecological poverty alleviation of firms and to strengthen the robustness of the results.

The importance of poverty alleviation assessment for officials is measured by whether the enterprise is located in the central and western provinces, and the number of state-level poor counties in the province. Firstly, *The Measures for Assessing the Effectiveness of Poverty Alleviation and Development Work of Provincial Party Committees and Governments* quantifies the effect of ecological poverty alleviation. The assessment method only applies to government officials in 22 central and western provinces (the 22 provinces (autonomous regions and municipalities directly under the central government) in the Midwest specifically include Hebei Province, Shanxi Province, Inner Mongolia Autonomous Region, Jilin Province, Heilongjiang Province, Anhui Province, Jiangxi Province, Henan Province, Hubei Province, Hunan Province, Guangxi Zhuang Autonomous Region, Hainan Province, Chongqing Municipality, Sichuan Province, Guizhou Province, Yunnan Province, Tibet Autonomous Region, Shaanxi Province, Gansu Province, Qinghai Province, Ningxia Hui Autonomous Region, and Xinjiang Uygur Autonomous Region). Local government officials in eastern provinces do not take part in the assessment. Therefore, compared with local government officials in eastern provinces, the assessment of the ecological poverty alleviation effectiveness of local government officials in central and western regions is more important in the comprehensive promotion evaluation.

Secondly, from the perspective of the scale of poverty alleviation objects, this paper uses the number of poverty-stricken counties at the national level to measure the importance of assessment of local government officials. Specifically, the greater the number of poverty-stricken counties, the more important the evaluation of ecological poverty alleviation effectiveness in the comprehensive promotion evaluation. There is some realistic basis for this measure. A poverty-stricken county at the national level is the standard set by the state to help poverty-stricken areas. Since China launched large-scale poverty alleviation in 1986, the central government has established a national poverty assessment method based on the per capita net income of each county. In 2016, 832 poverty-stricken counties at the national level were designated as key targets of poverty alleviation in *The Thirteenth Five-Year Plan for Poverty Alleviation*. Therefore, it is effective to measure the scale of poverty alleviation.

*Control* is a set of control variables. Referring to the previous literature related to the impact factors of corporate social responsibility [70,71,72,73], the following control variables are included: nature of firm ownership *Soe*, firm size *Size*, debt ratio *Lev*, return on assets *Roa*, cash ratio *Cashratio*, equity concentration *Shrhfd*, board size *Bsize*, and provincial fiscal deficit *Deficit*. In addition, the paper controls for annual fixed effects *φ* and provincial fixed effects *μ* [74,75]. The specific definitions and measures of the variables in the model are shown in Appendix A. Since *Povref_eco* is a dummy variable and *Input_eco* is a restricted dependent variable, logit regression and tobit regression are used, respectively [76,77], and the standard errors are adjusted for the clustering effect of firms. If Hypothesis 1 holds, *β*_1_ should be significantly positive.

## 4. Empirical Results

### 4.1. Descriptive Statistics

#### 4.1.1. Sample Distribution

Table 1 reflects the overall situation of ecological poverty alleviation among listed companies. The data show that overall, 381 samples are involved in ecological poverty alleviation, accounting for approximately 3.31% of the total sample. As *The Measures for Assessing the Effectiveness of Poverty Alleviation and Development Work of Provincial Party Committees and Governments* only apply to 22 provinces (autonomous regions and municipalities) directly under the central and western regions, the proportion of samples in the central and western regions participating in ecological poverty alleviation (7.09%) is higher than the proportion of samples in the eastern regions participating in ecological poverty alleviation (2.14%). This indicates that it is more common for firms in the central and western regions to participate in ecological poverty alleviation compared to those in the eastern regions. In addition, the proportion of samples in higher poverty areas participating in ecological poverty alleviation (7.35%) is higher than the proportion of samples in lower-poverty areas participating in ecological poverty alleviation (2.32%). The higher the level of poverty, the greater the importance of the poverty alleviation assessment on its government officials. Therefore, this phenomenon roughly suggests that enterprises’ participation in ecological poverty alleviation is likely to be related to the officials’ poverty alleviation effectiveness assessment.

#### 4.1.2. Descriptive Statistics for Key Variables

Table 2 presents the descriptive statistics of the main variables. It shows that nearly 3.31% of the sample of listed companies participate in ecological poverty alleviation. While the mean value of the amount of poverty alleviation input plus 1 taken as a logarithm is 0.0902, the maximum value is 4.7095, and the standard deviation is 0.5436, which indicates that the firms’ poverty alleviation input varies greatly. To describe the average level of poverty alleviation input of firms participating in ecological poverty alleviation, this paper calculates that the mean value of *Input_eco* without taking the natural logarithm is about RMB 0.322 million, and the maximum value is RMB 1.1 million, reflecting the importance that firms attach to ecological poverty alleviation. The proportion of samples in the middle and western areas is 23.62%, and there are, on average, five national poverty-stricken counties in the provinces to which the firms belong.

### 4.2. Univariate Analysis

Table 3 presents the differences in the ecological poverty alleviation of firms under the sample grouping. The result shows that both the ecological poverty alleviation possibility *Povref_eco* and the ecological poverty alleviation input level *Input_eco* are significantly greater for firms in the central and western regions than for those in the east, and firms in regions with high poverty are significantly greater than those with low poverty at the 1% confidence level. This result preliminarily verifies the main hypothesis and indicates that there is a positive correlation between enterprise participation in ecological poverty alleviation and official poverty alleviation assessment.

### 4.3. Basic Regression Results

Table 4 presents the effect of the officials’ poverty alleviation effectiveness assessment on firms’ ecological poverty alleviation, with regressions controlling for industry and year fixed effects and using firm-level clustering robust to standard errors to eliminate autocorrelation [78]. Columns (1) and (2) in Table 4 show a measure of importance of officials’ assessment poverty alleviation by whether the firm belongs to the *Midwest* province and it is found that the *Midwest*’s coefficient is significantly positive at the 1% confidence level for both the effect on whether the firm participates in eco-poverty alleviation and the number of firm eco-poverty inputs. Columns (3) and (4) in Table 4 show that the number of poor counties in each province is used to measure importance of officials’ pressure to assessment poverty alleviation. The results show that the coefficient of *Poor* is significantly positive at a 1% confidence level for both the effect on whether enterprises participate in ecological poverty alleviation and the amount of investment in ecological poverty alleviation. This indicates that the more important the assessment of poverty alleviation for officials in the province where the enterprise is located, the more likely the enterprise is to participate in ecological poverty alleviation and invest more money. This result supports Hypothesis 1.

### 4.4. Robust Test

#### 4.4.1. Propensity Score Matching

There is a systematic bias in the sample due to differences in firm characteristics variables when distinguishing firms in different poverty areas. Therefore, this paper uses a propensity score matching method [79] to ensure that there are no significant differences in firm characteristics and to test the impact of different assessment pressure regions on firms’ participation in ecological poverty alleviation. Based on the median number of poverty-stricken counties in province, the samples are divided into the group with more poverty-stricken counties and the group with fewer poverty-stricken counties. Firm size *Size*, debt ratio *Lev*, firm growth *Growth*, and industry dummy variables are used as matching variables for one-to-two nearest-neighbor replacement matching. The final samples of 1988 treated samples with more poverty-stricken counties and 2350 controlled samples with fewer poverty-stricken counties are obtained.

The characteristics of the treatment and control groups before and after propensity-score matching are shown in Appendix A. There are no significant group differences in firm size *Size*, debt ratio *Lev*, and firm growth *Growth*.

Subsequently, the matched treatment and control group samples are regressed according to Equation (1), with the results shown in Table 5. The coefficients of *Poor* are all significantly positive at the 1% confidence level, and this result remains consistent with the main test. It indicates that the conclusions of this paper still hold after eliminating the sample self-selection problem.

#### 4.4.2. Changing the Criteria for the Territorial Division of Firms

To avoid the measurement bias caused by inconsistency between the office address and registered address of a company, this paper measures the importance of assessment *Midwestw*, *Poorw*, and the regional fiscal deficit *Deficitw* faced by officials of the listed company’s location, using the office address as the attribution criterion for the province. Additionally, the impact of the importance of assessment on ecological poverty alleviation is retested. After changing the measure of the location of listed companies, the results are shown in Table 6 that the coefficients of *Midwestw* and *Poorw* are significantly positive and remain at the 1% level of significance for both the effect on *Povref_eco* and the effect on *Input_eco*. The results remain consistent with the results of the main test.

#### 4.4.3. Changing the Measurement of the Importance of Ecological Poverty Alleviation Assessment

To unify the statistical caliber of variables, this paper uses *Poornum*, the size of the poverty population, and *Pooratio*, the incidence of poverty in each province in that year, as proxy variables, from the National Bureau of Statistics. The results are shown in Columns (1)–(4) of Table 7. The coefficient of *Poornum* is positive at the 1% level of significance, and the coefficient of *Pooratio* is positive at the 1% level of significance for both the effect on *Povref_eco* and the effect on *Input_eco*. Once again, the robustness of the conclusions is confirmed.

#### 4.4.4. Changing the Measurement of Ecological Poverty Alleviation Inputs

I refer to the existing literature on the standardization of donations [80] and measure the level of firm ecological poverty alleviation inputs scaled by total assets in a lagged period *Povref_asset* and scaled by operating income in a lagged period *Povref_sale*. As shown in Table 8, the coefficients of *Midwest* and *Poor* are both significantly positive at the 10% confidence level after standardization of the input amount of the ecological poverty alleviation. Although the significance of the coefficients decreases, this result remains consistent with the main test.

### 4.5. Testing the Effects of Ecological Poverty Alleviation

Ecological poverty alleviation aims to improve the ecological environment of poverty-stricken areas while raising income levels by participating in ecological protection, ecological restoration projects and ecological industry development. Therefore, it is worth studying the effect of government mobilizing enterprises to participate in ecological poverty alleviation. Previous literatures on environmental problems in China mainly describe environmental pollution from air pollution, water pollution and solid pollution. Li et al. [81] proved that economic growth will improve air quality and water pollution in China using carbon dioxide, waste water, and waste solid emissions as different proxies of pollutants. In the same realm, He and Wang [82] used urban air quality data to show that pollution in China varies across economic and political structures, development strategies and environmental regulations.

This paper uses general industrial solid waste emissions, sulfur dioxide emissions in exhaust gas, and the total waste water emissions of each province to measure the local environmental pollution degree [81,82] to test the effect of enterprise ecological poverty alleviation investment to environmental improvement. The data are from the China Statistical Yearbook and China Environmental Statistical Yearbook, and the values of each variable are treated logarithmically. Column (1) in Table 9 tests the impact of enterprise ecological poverty alleviation on the general industrial solid waste emissions, while column (2) tests the impact of enterprise ecological poverty alleviation on local sulfur dioxide emission in waste gas, and column (3) tests the impact of enterprise ecological poverty alleviation on local waste water emissions. Due to the missing data of waste water emissions, the sample size involved in regression in column (3) is small. The results show that the coefficient of *Input_eco* is significantly negative at the 1% or 10% confidence level. It indicates that the enterprise ecological poverty alleviation investment will reduce local air pollution, water pollution and solid pollution, achieving environmental improvement. Therefore, the assessment mechanism driven by the enterprise ecological poverty alleviation is effective.

## 5. Conclusions and Discussion

### 5.1. Conclusions

Green development is an essential part of successful economic transformation in developing countries. The ecological poverty alleviation model can improve the ecological environment of poverty-stricken areas while increasing the income of the impoverished population. As a special form of poverty alleviation, firms are also actively involved. Therefore, it is worth looking into what motivates firms to participate in ecological poverty alleviation.

As local government officials are subject to strict ecological poverty alleviation effectiveness assessment, the assessment results are relevant to the officials’ career. Therefore, this paper hypothesizes that officials will mobilize enterprises to participate in ecological poverty alleviation because of the assessment. Based on a sample of A-share listed companies from 2016 to 2020, it is found that the firms in the central and western regions are more likely to participate in ecological poverty alleviation and invest more money in this jurisdiction compared to the eastern regions. Firms in the region with more poor counties are more likely to participate and invest more in ecological poverty alleviation. This paper further examines the impact of enterprise ecological poverty alleviation on environmental pollution and finds that enterprise participation in ecological poverty alleviation can indeed reduce solid pollution, water pollution and air pollution.

Firstly, this paper helps to expand the research on corporate environmental protection from the perspective of ecological poverty alleviation. Existing works in the literature mainly study the influencing factors of corporate environmental protection from the aspects of corporate reputation [17], public opinion pressure [18]. Additionally, existing works in the literature study the benefits of environmental protection to enterprises from the perspective of improving market performance [20,21] and reducing enterprise risk [22]. However, there are few works in the literature on ecological poverty alleviation as a special way of environmental protection. This paper directly studies a new and special environmental protection behavior, which can enrich and expand relevant research on green investment from the micro level.

Secondly, this paper is helpful to expand the literature related to government–enterprise interaction from the perspective of assessment of government officials. As for the relevant literature on government–enterprise interaction, the existing literature mainly identifies the policy burden of state-owned enterprises through government intervention [28,29] and shows that firms help politicians achieve their personal political goals and politicians also give benefits back [30,31,32]. The process of government–enterprise interaction studied in this paper is based on the assessment of local government officials as a logical starting point. This is clearer about the role of the assessment mechanism of local government officials than previous literatures. Therefore, this paper provides new evidence for relevant literature on government–enterprise interaction from the perspective of government officials’ assessment.

Finally, this paper helps to enrich the relevant literature on the promotion incentives for government officials from the perspective of ecological poverty alleviation. Existing works on promotion incentives for government officials mostly focus on the impact of promotion incentives on regional economic growth [35,36], bank bankruptcy [37,38], investment scale [40] and firm performance [41,44]. However, few works study the influence of the promotion incentive mechanism on corporate social activities. This paper indicates that the official assessment can stimulate not only the economic decisions, but also non-economic decisions. Therefore, this paper enriches the relevant literature on promotion incentives of government officials from the perspective of ecological poverty alleviation.

### 5.2. Practical Implications

Firstly, this research has implications for improving environmental governance. In a situation where the government, market, and social forces cooperate in environmental governance, the government is no longer the sole ‘producer’ of performance but needs a ‘cooperative production’ process. In addition to the government’s own investment of resources in ecological poverty alleviation, the government also needs firms to invest with diversified resources as a guarantee. Therefore, the ecological support of enterprises is very important in environmental governance.

Secondly, the research has reference value and implications for improving the appraisal and assessment of government officials. The introduction of a promotion appraisal mechanism for government officials gives the government a strong incentive to drive enterprises to invest resources in ecological poverty alleviation work, and the resources are interdependent. On this basis, the government also needs to increase the mobilization of firm power to participate in ecological poverty alleviation by giving them the necessary policy support, external environmental support, and service support.

### 5.3. Limitations and Future Research

Using a sample of listed companies, this paper empirically tests and finds that the government’s poverty assessment drives corporate participation in ecological poverty alleviation. Although the results pass a series of robustness tests, there are certain limitations. In the future, we can further discuss the following limitations.

First, there are limitations in the ecological poverty alleviation information disclosed by the annual reports of listed companies, which makes it impossible to further obtain the specific project content of enterprises’ participation in ecological poverty alleviation, to further discuss how to optimize the ecological poverty alleviation mode of enterprises. In the future, case studies and questionnaire surveys can be used to investigate the specific mode of enterprise ecological poverty alleviation, the efficiency of fund use and the effect of ecological poverty alleviation. We can further study the impact of ecological poverty alleviation on enterprise performance.

Second, this paper only considers the forms of ecological poverty alleviation defined by enterprises. In fact, in reality, other non-ecological forms of poverty alleviation, such as industrial poverty alleviation, relocation and employment poverty alleviation, may also achieve the purpose of ecological protection. For example, enterprises should standardize and guide the planting and breeding industry and turn ecological environmental advantages into driving forces for industrial development. Enterprises can convert farmland to forest through relocation, and enterprises can improve the ecological environment while increasing the employment of rural population. Therefore, future research can combine the indirect impact of different forms of poverty alleviation on ecological and environmental protection.

Thirdly, this paper only considers the enterprise ecological poverty alleviation under the scenario of China. Whether this ecological poverty alleviation model is universal and can be used for reference by other countries needs further study. Future studies can make use of multi-country samples to compare and analyze the specific characteristics, advantages and disadvantages of ecological poverty alleviation in various countries and the implementation effects, as well as analyzing the applicability of China’s ecological poverty alleviation model.

## Figures and Tables

**Table 1:** Sample distribution.

Group	Participation in Ecological Poverty Alleviation		No Participation in Ecological Poverty Alleviation	
	N	Proportion (%)	N	Proportion (%)
Overall	381	3.31	11,146	96.69
Midwest Region	193	7.09	2530	92.91
Eastern Region	188	2.14	8616	97.86
High poverty	166	7.35	2093	92.65
Low poverty	215	2.32	9053	97.68

**Table 2:** Descriptive statistics for key variables.

Variable	N	Mean	sd	Min	p25	p50	p75	Max
Povref_eco	11,527	0.0331	0.1788	0.0000	0.0000	0.0000	0.0000	1.0000
Input_eco	11,527	0.0902	0.5436	0.0000	0.0000	0.0000	0.0000	4.7095
Midwest	11,527	0.2362	0.4248	0.0000	0.0000	0.0000	0.0000	1
Poor	11,527	0.0563	0.1404	0.0000	0.0000	0.0000	0.0000	0.88
Soe	11,527	0.2397	0.4269	0.0000	0.0000	0.0000	0.0000	1
Size	11,527	21.9881	1.1663	19.5950	21.1525	21.8515	22.6523	26.1024
Lev	11,527	0.3966	0.1988	0.0523	0.2357	0.3822	0.5359	0.933
Roa	11,527	0.0355	0.0802	−0.5304	0.0151	0.0401	0.0714	0.2479
Cashratio	11,527	0.8226	1.1613	0.0206	0.2047	0.4197	0.9158	8.9238
Shrhfd	11,527	0.1479	0.1012	0.0128	0.0706	0.1224	0.2011	0.5013
Bsize	11,527	2.2529	0.2530	1.6094	2.0794	2.1972	2.3979	2.8904
Deficit	11,527	7.8751	0.5296	6.2399	7.4535	8.0079	8.2686	8.8447

All continuous variables at firm level are winsorized at the 1% and 99% by year.

**Table 3:** Univariate analysis.

Group Standard	Group	Povref_eco			Input_eco		
		N	Mean	Difference (*t*-Value)	N	Mean	Difference (*t*-Value)
whether poverty alleviation assessment apply	Midwest	2723	0.0709	0.0495 12.7205 ***	2723	0.1843	0.1232 10.3833 ***
	East	8804	0.0214		8804	0.0611	
importance of poverty alleviation assessment	High poverty	2259	0.0735	0.0503 12.0620 ***	2259	0.1871	0.1205 9.4878 ***
	Low poverty	9268	0.0232		9268	0.0666	

*** denotes significance levels of 0.01.

**Table 4:** The impact of the importance of poverty alleviation assessment on enterprise ecological poverty alleviation.

Variable	(1)	(2)	(3)	(4)
	Povref_eco	Input_eco	Povref_eco	Input_eco
Midwest	0.8936 ***	1.9099 ***		
	(3.9152)	(4.6716)		
Poor			2.3658 ***	4.5618 ***
			(4.3165)	(4.6114)
Soe	0.4654 **	0.8664 **	0.5272 **	1.0056 **
	(2.0969)	(2.1613)	(2.3729)	(2.5148)
Size	0.9491 ***	1.8650 ***	0.9235 ***	1.8171 ***
	(9.6030)	(10.8283)	(9.5353)	(10.7643)
Lev	−0.4641	−0.8186	−0.2942	−0.5627
	(−0.6836)	(−0.6494)	(−0.4486)	(−0.4579)
Roa	0.5552	1.8213	0.7334	1.8566
	(0.4529)	(0.7868)	(0.6019)	(0.8073)
Cashratio	−0.0441	−0.1264	−0.0342	−0.1011
	(−0.4277)	(−0.6549)	(−0.3320)	(−0.5307)
Shrhfd	1.4711	2.9438	1.3525	2.6513
	(1.5351)	(1.6434)	(1.4306)	(1.5005)
Bsize	−0.1939	−0.5269	−0.1746	−0.5336
	(−0.6491)	(−0.9499)	(−0.5866)	(−0.9707)
Deficit	0.2584	0.2864	0.2896	0.4221
	(1.0429)	(0.6889)	(1.2025)	(1.0369)
_cons	−27.6206 ***	−52.8375 ***	−27.1475 ***	−52.5975 ***
	(−8.3377)	(−9.6013)	(−8.1590)	(−9.4798)
Year FE	Yes	Yes	Yes	Yes
Industry FE	Yes	Yes	Yes	Yes
pseudo R-sq	0.334	0.250	0.331	0.246
N	9712	11,527	9712	11,527

*** and ** denote significance levels of 0.01and 0.05, respectively. Logit regressions correspond to z-values in parentheses, and tobit regressions correspond to *t*-values in parentheses. Standard errors are adjusted for firm clustering effects.

**Table 5:** Robustness tests: sample self-selection.

Variable	(1)	(2)
	Povref_eco	Input_eco
Poor	2.5743 ***	4.7120 ***
	(3.9657)	(4.4050)
Soe	0.3538	0.5481
	(1.3840)	(1.2049)
Size	0.8715 ***	1.6537 ***
	(7.3283)	(8.4285)
Lev	0.0821	0.2385
	(0.1103)	(0.1754)
Roa	1.8105	4.1662
	(1.2178)	(1.5494)
Cashratio	−0.0814	−0.2105
	(−0.5970)	(−0.8491)
Shrhfd	0.5886	0.9660
	(0.4979)	(0.4627)
Bsize	−0.2094	−0.4422
	(−0.5449)	(−0.6558)
Deficit	0.3506	0.5556
	(1.0879)	(1.0875)
_cons	−26.2579 ***	−49.2722 ***
	(−6.6143)	(−7.9742)
Year FE	Yes	Yes
Industry FE	Yes	Yes
pseudo R-sq	0.306	0.234
N	3523	4338

*** denotes significance levels of 0.01. Logit regressions correspond to z-values in parentheses, and tobit regressions correspond to *t*-values in parentheses. Standard errors are adjusted for firm clustering effects.

**Table 6:** Robustness tests: attribution criteria with office address.

Variable	(1)	(2)	(3)	(4)
	Povref_eco	Input_eco	Povref_eco	Input_eco
Midwestw	0.9229 ***	1.9286 ***		
	(4.0659)	(4.7121)		
Poorw			2.3086 ***	4.4009 ***
			(4.2485)	(4.3977)
Size	0.9901 ***	1.9317 ***	0.9670 ***	1.8982 ***
	(10.1541)	(11.4274)	(10.2167)	(11.4898)
Lev	−0.2809	−0.5026	−0.0830	−0.2100
	(−0.4249)	(−0.4080)	(−0.1294)	(−0.1738)
Roa	0.4063	1.5429	0.5896	1.5733
	(0.3438)	(0.6919)	(0.4991)	(0.7061)
Cashratio	−0.0348	−0.1102	−0.0210	−0.0787
	(−0.3420)	(−0.5834)	(−0.2072)	(−0.4236)
Shrhfd	1.6427 *	3.3007 *	1.5771 *	3.1043 *
	(1.7677)	(1.9073)	(1.7195)	(1.8160)
Bsize	−0.0884	−0.2635	−0.0295	−0.1799
	(−0.2921)	(−0.4648)	(−0.0976)	(−0.3182)
Deficitw	0.2464	0.3158	0.3046	0.5024
	(1.0338)	(0.7791)	(1.3147)	(1.2693)
_cons	−28.4036 ***	−54.6466 ***	−28.2108 ***	−55.1705 ***
	(−8.6301)	(−9.9026)	(−8.5690)	(−9.9182)
Year FE	Yes	Yes	Yes	Yes
Industry FE	Yes	Yes	Yes	Yes
pseudo R-sq	0.330	0.247	0.326	0.243
N	9712	11,527	9712	11,527

*** and * denote significance levels of 0.01 and 0.1, respectively. Logit regressions correspond to z-values in parentheses, and tobit regressions correspond to *t*-values in parentheses. Standard errors are adjusted for firm clustering effects.

**Table 7:** Robustness tests: changing the measurement of importance of assessment.

Variable	(1)	(2)	(3)	(4)
	Povref_eco	Input_eco	Povref_eco	Input_eco
Poornum	0.3848 ***	0.7331 ***		
	(2.7914)	(3.0572)		
Pooratio			0.1028 **	0.2345 ***
			(2.2819)	(2.8205)
Soe	0.5478 **	1.0026 **	0.5406 **	0.9932 **
	(2.3290)	(2.4203)	(2.2862)	(2.3821)
Size	0.8216 ***	1.5609 ***	0.8263 ***	1.5830 ***
	(7.5991)	(8.3827)	(7.6251)	(8.4336)
Lev	0.1741	0.6190	0.1062	0.3854
	(0.2549)	(0.4941)	(0.1539)	(0.3061)
Roa	1.4546	3.9440	1.3262	3.7580
	(1.0779)	(1.5324)	(0.9994)	(1.4765)
Cashratio	0.0332	0.0361	0.0263	0.0170
	(0.2810)	(0.1628)	(0.2216)	(0.0762)
Shrhfd	1.6966 *	3.3477 *	1.6304 *	3.2527 *
	(1.7468)	(1.8656)	(1.6871)	(1.8087)
Bsize	0.0089	−0.0862	−0.0106	−0.1152
	(0.0265)	(−0.1450)	(−0.0315)	(−0.1929)
Deficit	0.5069 *	0.7833 *	0.6913 ***	1.1023 **
	(1.8625)	(1.7081)	(2.5824)	(2.4899)
_cons	−26.9328 ***	−50.5075 ***	−28.5009 ***	−53.8464 ***
	(−7.1941)	(−8.3350)	(−7.7963)	(−9.0303)
Year FE	Yes	Yes	Yes	Yes
Industry FE	Yes	Yes	Yes	Yes
pseudo R-sq	0.313	0.236	0.311	0.235
N	7291	8768	7291	8768

***, **, and * denote significance levels of 0.01, 0.05, and 0.1, respectively. Logit regressions correspond to z-values in parentheses, and tobit regressions correspond to *t*-values in parentheses. Standard errors are adjusted for firm clustering effects.

**Table 8:** Robustness tests: changing the measurement of ecological poverty alleviation inputs.

Variable	(1)	(2)	(3)	(4)
	Povref_asset	Povref_asset	Povref_sale	Povref_sale
Midwest	14.9279 *		23.7309 *	
	(1.8056)		(1.8056)	
Poor		34.3875 *		54.6652 *
		(1.8178)		(1.8178)
Soe	7.3219	8.3566 *	11.6385	13.2834 *
	(1.6414)	(1.7085)	(1.6413)	(1.7084)
Size	12.3344 **	11.9336 **	19.6080 **	18.9707 **
	(1.9907)	(1.9921)	(1.9908)	(1.9921)
Lev	0.6302	2.5085	1.0009	3.9869
	(0.0675)	(0.2646)	(0.0675)	(0.2645)
Roa	10.9163	11.1117	17.3253	17.6321
	(0.6411)	(0.6582)	(0.6400)	(0.6569)
Cashratio	−0.6010	−0.4211	−0.9554	−0.6694
	(−0.4092)	(−0.2927)	(−0.4091)	(−0.2926)
Shrhfd	26.6023	24.0436	42.2898	38.2224
	(1.3925)	(1.3382)	(1.3925)	(1.3382)
Bsize	−6.6696	−6.7045	−10.6008	−10.6559
	(−1.1001)	(−1.1098)	(−1.1000)	(−1.1096)
Deficit	1.2443	2.3926	1.9780	3.8034
	(0.4331)	(0.8383)	(0.4331)	(0.8382)
_cons	−361.6119 **	−359.5072 **	−574.8584 **	−571.5113 **
	(−2.0530)	(−2.0502)	(−2.0530)	(−2.0502)
Year FE	Yes	Yes	Yes	Yes
Industry FE	Yes	Yes	Yes	Yes
pseudo R-sq	0.149	0.147	0.141	0.138
N	11,527	11,527	11,526	11,526

**, and * denote significance levels of 0.05, and 0.1, respectively. Logit regressions correspond to z-values in parentheses, and tobit regressions correspond to *t*-values in parentheses. Standard errors are adjusted for firm clustering effects.

**Table 9:** The effects of ecological poverty alleviation.

Variable	(1)	(2)	(3)
	SolidWaste	Dioxide	WasteWater
Input_eco	−0.0791 ***	−0.0531 ***	−0.0372 *
	(−3.5585)	(−2.7530)	(−1.7143)
Size	−0.0167	−0.0239 **	−0.0115
	(−1.2347)	(−2.1623)	(−0.9983)
Lev	0.1441 *	0.0973	−0.3382 ***
	(1.6918)	(1.4328)	(−4.5244)
Roa	0.1738	0.0547	0.3245 ***
	(1.5761)	(0.5966)	(3.0031)
Cashratio	−0.0234 **	−0.0206 **	−0.0401 ***
	(−2.2392)	(−2.5068)	(−3.8089)
Shrhfd	−0.1490	−0.0296	0.1806 *
	(−1.2621)	(−0.2960)	(1.7361)
Bsize	0.0899 **	0.0385	−0.1166 ***
	(2.2702)	(1.1679)	(−3.3337)
Deficit	1.5237 ***	1.4755 ***	0.7455 ***
	(61.1830)	(75.0264)	(32.1157)
_cons	−2.8803 ***	−7.7875 ***	6.5105 ***
	(−7.8249)	(−26.2382)	(20.0854)
Year FE	Yes	Yes	Yes
Industry FE	Yes	Yes	Yes
adj. R-sq	0.595	0.667	0.362
N	11527	11527	8133

***, **, and * denote significance levels of 0.01, 0.05, and 0.1, respectively. Logit regressions correspond to z-values in parentheses, and tobit regressions correspond to *t*-values in parentheses. Standard errors are adjusted for firm clustering effects.

## Data Availability

The data presented in this study are available on request from China Stock Market and Accounting Research Database (CSMAR) database, the National Bureau of Statistics and the China Poverty Alleviation Database.

## Appendix A

1. The Definition of Poverty

The first goal of the United Nations sustainable development is to eliminate all forms of poverty around the world. Poverty, however, is a complex concept. Poverty is not only a lack of income and resources that makes it difficult to make ends meet. It is also manifested in hunger and malnutrition, inadequate access to education and other basic public services, social discrimination and exclusion, and lack of participation in decision making. The Decision of the CPC Central Committee and The State Council on Winning the Tough Battle against Poverty, issued in November 2015, further clarified that by 2020, China will ensure the basic living conditions, education, basic medical care and housing of the poor population, and ensure that the per capita disposable income of farmers in poor areas increases by more than the national average This is China’s effort to eliminate poverty.

2. The Definition of Variables

**Table A1:** Definition of variables.

Variable Name	Variable Symbols	Variable Measurement
Ecological poverty alleviation	Povref_eco	The variable equals 1 if the company is involved in ecological poverty alleviation in the t year and 0 if it is not involved in any form of poverty alleviation.
	Input_eco	The total amount of the firm’s ecological poverty alleviation input is added by 1 and then taken as the natural logarithm. If not involved in ecological poverty alleviation, the total amount of ecological poverty alleviation input is 0.
The importance of ecological poverty alleviation assessment	Poor	Number of national-level poverty-stricken counties by province for the year, combined national-level poverty-stricken counties and sub-counties in special contiguous areas, in hundreds.
	Poornum	Size of the poor population by province for the year, in millions.
	Pooratio	Poverty incidence by province for the year, in %.
Nature of firm ownership	Soe	If the ultimate controller is a state-owned legal person, a state-owned government agency, and a state-controlled enterprise, such as an institution or autonomous organization, then the value of Soe equals 1 for state-owned enterprises; otherwise, it is 0.
Firm size	Size	Natural logarithm of total assets at the end of the period.
Debt ratio	Lev	Lev = total liabilities at end of period/total assets at end of period.
Return on assets	Roa	Roa = net profit/total assets.
Cash ratio	Cashratio	(Monetary funds + financial assets held for trading + notes receivable)/total current liabilities.
Equity concentration	Shrhfd	Sum of the squares of the top five shareholders’ shareholdings.
Board size	Bsize	Natural logarithm of the total number of directors on the board.
Provincial fiscal deficit	Decifit	Natural logarithm of fiscal deficits for the year by province.

3. Correlation Coefficient Test

In this paper, the Pearson correlation coefficient analysis is conducted for the main regression variables, and the result in Table A2 shows that *Povref_eco* and *Input_eco* have a positive relationship with *Midwest* and *Poor*, which initially proves the hypothesis. Except for a large correlation coefficient between the indicators measuring ecological poverty alleviation, the correlation coefficients between the other variables are less than 0.6, so there is no serious co-linearity.

**Table A2:** Correlation coefficient test.

Panel A: Correlation Coefficient of Povref_eco to Size						
Variable	Povref_eco	Input_eco	Midwest	Poor	Soe	Size
Povref_eco	1					
Input_eco	0.897 ***	1				
Midwest	0.118 ***	0.096 ***	1			
Poor	0.085 ***	0.059 ***	0.721 ***	1		
Soe	0.160 ***	0.128 ***	0.164 ***	0.117 ***	1	
Size	0.251 ***	0.249 ***	0.042 ***	0.002	0.328 ***	1
Lev	0.118 ***	0.111 ***	0.088 ***	0.054 ***	0.265 ***	0.480 ***
Roa	0.002	0.007	−0.050 ***	−0.036 ***	−0.073 ***	−0.023
Cashratio	−0.056 ***	−0.054 ***	−0.019	−0.016	−0.106 ***	−0.276 ***
Shrhfd	0.079 ***	0.060 ***	−0.025	−0.026	0.145 ***	0.116 ***
Bsize	0.071 ***	0.066 ***	0.091 ***	0.069 ***	0.258 ***	0.222 ***
Deficit	0.036 ***	0.031 *	0.320 ***	0.245 ***	−0.061 ***	−0.026
**Panel B: Correlation Coefficient of Lev to Deficit**						
	**Lev**	**Roa**	**Cashratio**	**Shrhfd**	**Bsize**	**Deficit**
Lev	1					
Roa	−0.325 ***	1				
Cashratio	−0.556 ***	0.188 ***	1			
Shrhfd	−0.003	0.173 ***	0.026	1		
Bsize	0.166 ***	−0.114 ***	−0.092 ***	−0.041 ***	1	
Deficit	0.012	−0.019	−0.032 **	−0.041 ***	−0.026	1

***, **, and * denote significance levels of 0.01, 0.05, and 0.1, respectively.

4. Propensity Score Matching Balance Test

The characteristics of the treatment and control groups before and after propensity-score matching are shown in Table A3. There are no significant group differences in firm size *Size*, debt ratio *Lev*, and firm growth *Growth*.

**Table A3:** Propensity score matching balance test.

	Unmatched	Mean			%reduct	*t*-Test	
Variable	Matched	Treated	Control	%bias	|bias|	*t*	*p* > |*t*|
Size	U	22.133	22.051	7	40.4	2.75	0.006
	M	22.133	22.084	4.2		1.29	0.197
Growth	U	0.21895	0.19967	3.8	−48.9	1.57	0.116
	M	0.21895	0.24765	−5.7		−1.63	0.104
Lev	U	0.43957	0.39605	21.3	80.9	8.42	0
	M	0.43957	0.43126	4.1		1.27	0.205
